# Cardiac Involvement in Eosinophilic Granulomatosis with Polyangiitis

**DOI:** 10.1007/s11886-025-02258-z

**Published:** 2025-07-09

**Authors:** Mukunthan Srikantharajah, Deepa Gopalan, Harold Wilson-Morkeh, Salman Siddiqui, Stephen P. McAdoo, Taryn Youngstein

**Affiliations:** 1 Vasculitis Centre, Department of Immunology and Inflammation, Imperial College London, London, UK; 2 Department of Renal Medicine, Hammersmith Hospital, London, UK; 3 Vasculitis Centre, Department of Imaging Sciences, Imperial College London, London, UK; 4 Department of Non-Invasive Cardiac Imaging, Hammersmith Hospital, London, UK; 5 Vasculitis Centre, National Heart and Lung Institute, Imperial College London, London, UK; 6 Department of Rheumatology, Hammersmith Hospital, London, UK; 7 Department of Respiratory Medicine, Hammersmith Hospital, London, UK

**Keywords:** Eosinophilic granulomatosis with polyangiitis, Vasculitis, Cardiovascular, Cardiac magnetic resonance

## Abstract

**Purpose of Review:**

This review highlights recent advances in the pathophysiology, diagnosis, and treatment of cardiac disease in patients with Eosinophilic granulomatosis with polyangiitis (EGPA). We outline a diagnostic approach to facilitate early identification of affected patients.

**Recent Findings:**

Recent advancements in diagnostic techniques including cardiac magnetic resonance (CMR) have improved recognition of cardiac disease in patients with EGPA. CMR has demonstrated a high prevalence of cardiac abnormalities, even in the absence of clinical symptoms, electrocardiographic or echocardiographic findings.

**Summary:**

Cardiac disease is a major cause of mortality in patients with EGPA, accounting for around 50% of disease-related deaths. However, due to the lack of standardised screening and diagnostic criteria, the true incidence remains unknown. Systemic immunosuppressive therapy is warranted to prevent acute complications as well as mitigate the long-term impact of chronic inflammation and tissue damage. Given the challenges in early detection and the prognostic significance of cardiac involvement, we recommend including CMR in the baseline evaluation of all EGPA patients at diagnosis.

## Introduction

Eosinophilic granulomatosis with polyangiitis (EGPA, formerly Churg-Strauss syndrome) is a chronic inflammatory disorder characterized by asthma, chronic rhinosinusitis, blood and tissue eosinophilia, and small-to-medium vessel necrotising vasculitis [1]. Anti-neutrophil cytoplasm antibodies (ANCA) are present in 30–40% of cases and typically directed against myeloperoxidase (MPO) [2, 3], thus the disease is classified as an ANCA-associated pauci-immune vasculitis by the Chapel Hill Consensus Nomenclature of Vasculitides [4].

The natural history of EGPA is classically described as three overlapping phases: an initial prodromal stage with ‘allergic’ features such as atopy, asthma, and rhinosinusitis; an eosinophilic stage characterized by peripheral blood eosinophilia and clinical manifestations arising from eosinophilic tissue infiltration; and a third stage involving necrotizing vasculitis of small-to-medium-sized blood vessels [5]. Significant heterogeneity is observed in the disease and not all patients exhibit overt vasculitis [6].

Cardiac disease in patients with EGPA is common, typically occurring during the latter two (eosinophilic and vasculitic) phases, at reported frequencies ranging between 16 and 92% in different cohorts [7, 8]. As such, EGPA is among the most common systemic vasculitides to affect the heart. However, in the absence of verified screening and diagnostic criteria, there is no universal definition for cardiac involvement in EGPA and thus the true incidence remains unknown [9].

**Fig. 1:**
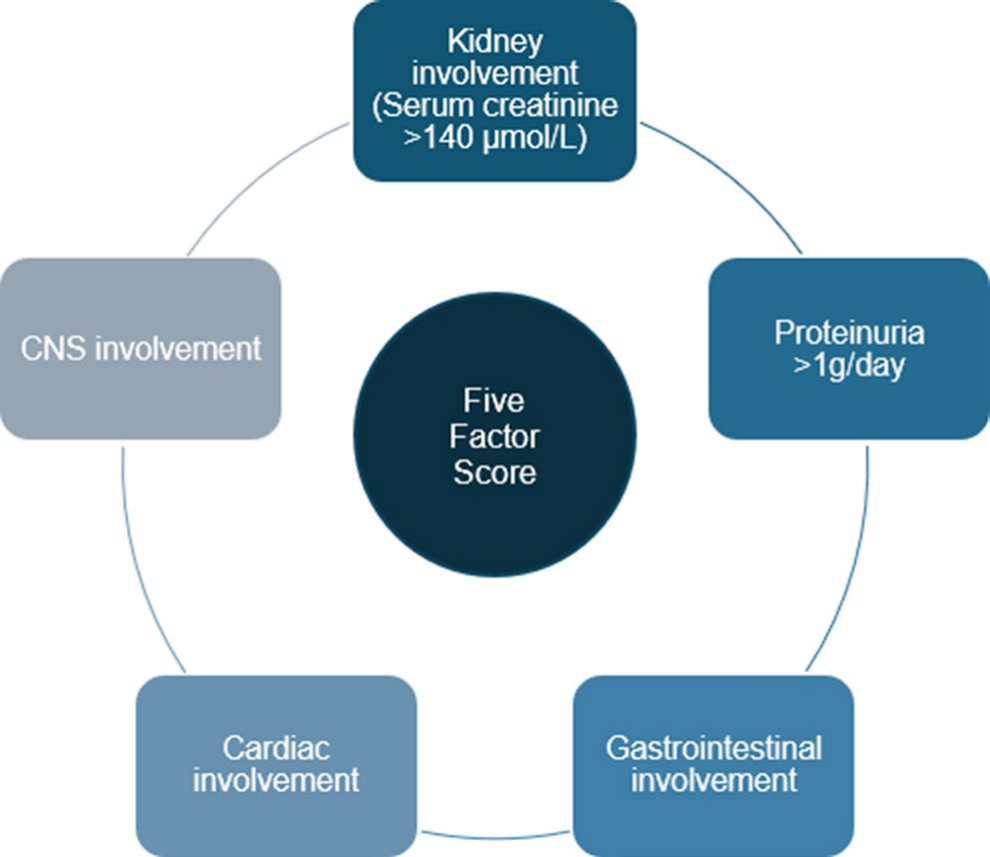
Original five factor score. The five factor Score (FFS) predicts the risk of mortality in patients with an established diagnosis of EGPA. It consists of five factors associated with shortened survival: cardiac involvement, CNS involvement, kidney involvement (serum creatinine > 140 µmol/L), proteinuria > 1 g/day and gastrointestinal involvement. This has been subsequently revised to include age > 65 years as a poor prognostic factor, ENT involvement as a favourable prognostic factor, and CNS involvement no longer included in the score [10, 99]. Abbreviations: CNS, Central Nervous System; FFS, Five Factor Score; ENT, Ear Nose Throat

Cardiomyopathy is recognised as a poor prognostic factor in all systemic vasculitides (alongside kidney insufficiency, proteinuria, gastrointestinal and central nervous system involvement), as defined by the original Five Factor Score (FFS) (Fig. 1) [10]. It is a major cause of mortality in patients with EGPA, with 50% of deaths being related to cardiac disease. EGPA-related cardiomyopathy increases the mortality risk by four-fold, and five-year survival decreases from 91.6 to 78.2% in the presence of myocardial involvement [11].

Diagnosing cardiac involvement in EGPA patients is challenging. Disease presentation can be insidious, and many patients do not display typical cardiac symptoms or investigation findings (such as elevated serum cardiac biomarkers (troponin, B-type natriuretic peptide (BNP) and N-terminal pro-BNP (NT-proBNP)), electrocardiographic (ECG) or echocardiographic abnormalities indicative of cardiac disease. This has likely led to under-recognition of this condition in the past [5]. There has been a recent increase in awareness of cardiac disease in EGPA, attributable to advances in diagnostic techniques including cardiac magnetic resonance (CMR) imaging, and long-term follow up of large, multicentre cohorts confirming poor outcomes. This review presents recent advances in the pathophysiology, diagnosis, and treatment approaches to cardiac disease in EGPA patients.

## Pathophysiology

**Fig. 2:**
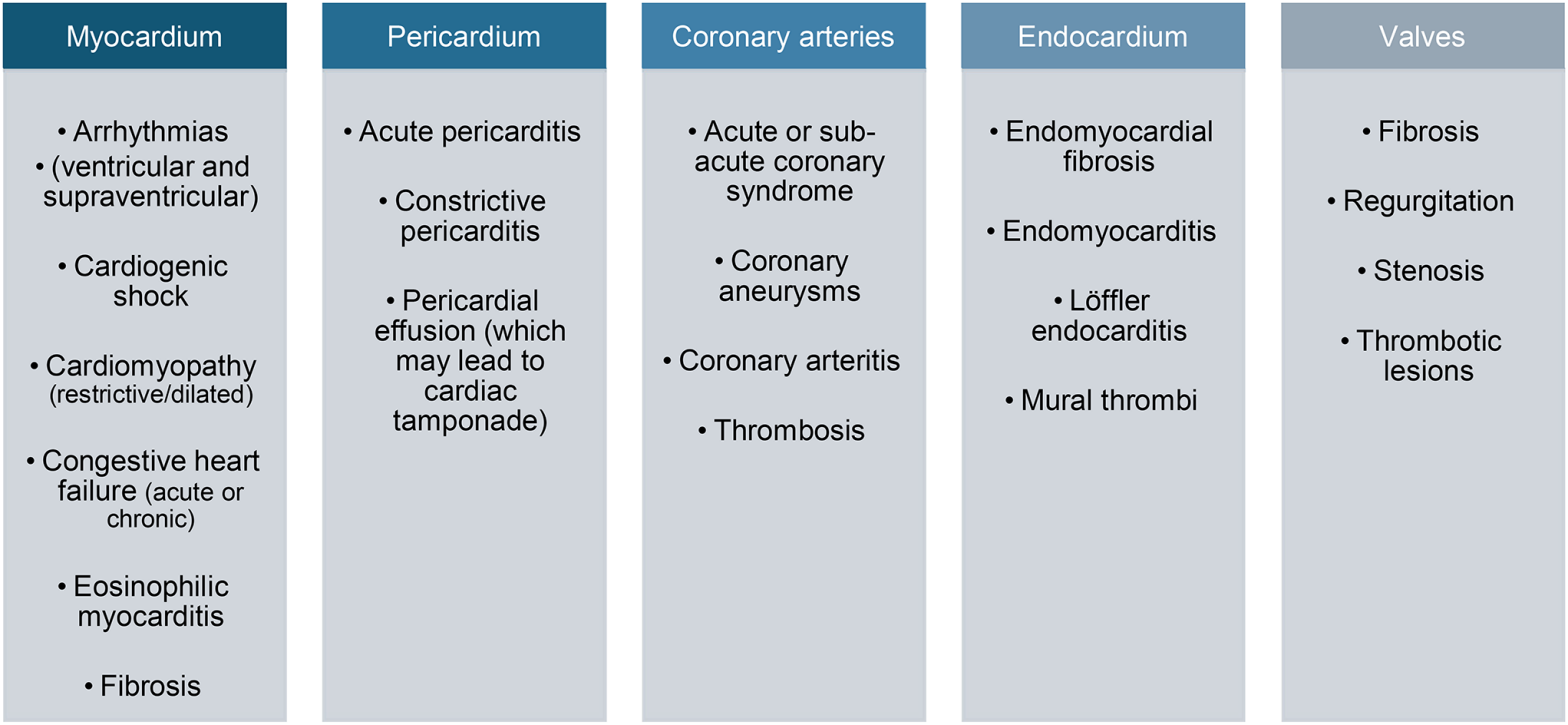
Cardiac involvement in EGPA. Cardiac disease in EGPA can affect the myocardium, pericardium, coronary arteries, endocardium and valvular structures Data derived from [8, 12, 22, 30, 30, 38, 48]

Cardiac involvement in EGPA can affect the myocardium, pericardium, coronary arteries, endocardium and valvular structures (Fig. 2).

EGPA is typically considered a T_H_2 mediated disease with blood and tissue eosinophilia representing the cornerstone of diagnosis [12]. Cardiac disease can be understood as progressing through the following stages, all of which can co-exist [13]. 

### Tissue Necrosis

Eosinophilic infiltration of myocardial tissue (i.e. cardiomyocytes, endothelial cells, other interstitial cells) and degranulation of eosinophils leads to cellular damage via release of toxic granule proteins, such as eosinophilic cationic protein (ECP) and major basic protein (MBP) that contribute directly to inflammation, tissue damage, and cell death [14, 15]. Elevated expression of the ligand’s granulocyte-macrophage colony-stimulating factor (GM-CSF), interlukin-3 (IL-3), IL-5 and their receptors is also observed. Animal models in EGPA are lacking but there is in vitro evidence to suggest that cardiotoxicity is mediated by ECP through alteration of the membrane sodium permeability of cardiomyocytes and the inhibition of mitochondrial respiration [12].

### Thrombosis

Eosinophils contribute to microvascular damage and hyperactivation of endogenous coagulation systems, releasing eosinophil granule proteins involved in blood coagulation as tissue factors, promoting a hypercoagulable state. Prothrombotic changes can result from ECP- and MBP- induced alterations in the coagulation cascade, as well as aberrant eosinophil-derived reactive oxygen species (ROS) production [12]. Eosinophils can generate thrombin and induce tissue factor exposure on endothelial cells, leading to increased platelet adhesion to the vascular wall and thrombus development. Furthermore, intravascular eosinophil activation also induces formation of eosinophil extracellular traps (EETs) promoting additional thrombosis [16, 17]. The eosinophils’ ability to induce a prothrombotic environment on endothelium translates to an increased risk of arterial and venous thrombosis.

### Fibrosis

Fibrosis can affect both valve and heart wall structures [13]. Eosinophil granule proteins promote fibrosis by releasing transforming growth factor- β, IL-1α, and IL-1β, suggesting that myocardial fibrosis occurs as part of the primary disease pathogenesis as well as the sequelae of longstanding inflammation and tissue damage (and thus can occur in absence of significant inflammatory disease) [14]. In fact, the presence of eosinophilic infiltrates and granule proteins has been documented in fibrotic tissue from endomyocardial biopsies of patients with EGPA [18, 19].

### Vasculitis

Whilst the pathogenic effects of eosinophils in cardiac disease are understood, the role of neutrophils is less well described, however a ‘vasculitic’ component is deemed to play a role in its pathology. For example, in tissue samples of EGPA explanted hearts (from patients who underwent cardiac transplantation), 67% showed classical signs of active vasculitis such as neutrophil-rich small-vessel infiltration, fibrinoid necrosis, arteritis and thrombosis suggesting a neutrophil-induced vasculitis component to cardiac disease [20]. Although there is limited understanding of neutrophil-eosinophilic interactions in EGPA cardiac disease, we speculate that neutrophils may infiltrate tissue because of eosinophil-derived cytokine production. Furthermore, given the shared genetic basis of MPO-ANCA-associated vasculitis (AAV) and MPO-positive EGPA, neutrophilic vasculitis may also arise as a primary phenomenon [12].

## Presentation

Cardiac involvement is often identified at the initial presentation of EGPA. In a retrospective study by Sartorelli et al. (2022), involving 70 patients with EGPA-associated cardiomyopathy, 58 patients (83%) were diagnosed with cardiomyopathy at the time of EGPA onset [9]. Typical signs and symptoms include chest pain, palpitations, hypotension, arrhythmias (ranging from sinus tachycardia to complex ventricular arrhythmias) and cardiogenic shock [9], and instances of sudden cardiac death are recognised [21]. However, patients may not report specific cardiac symptoms. A retrospective review found 11.1% of patients were asymptomatic [5], and even in symptomatic patients, symptom burden may correlate poorly with the extent of disease [5]. Thus, screening for cardiac disease is recommended in all patients, although the optimum screening approach is not yet defined.

## Investigations

**Fig. 3:**
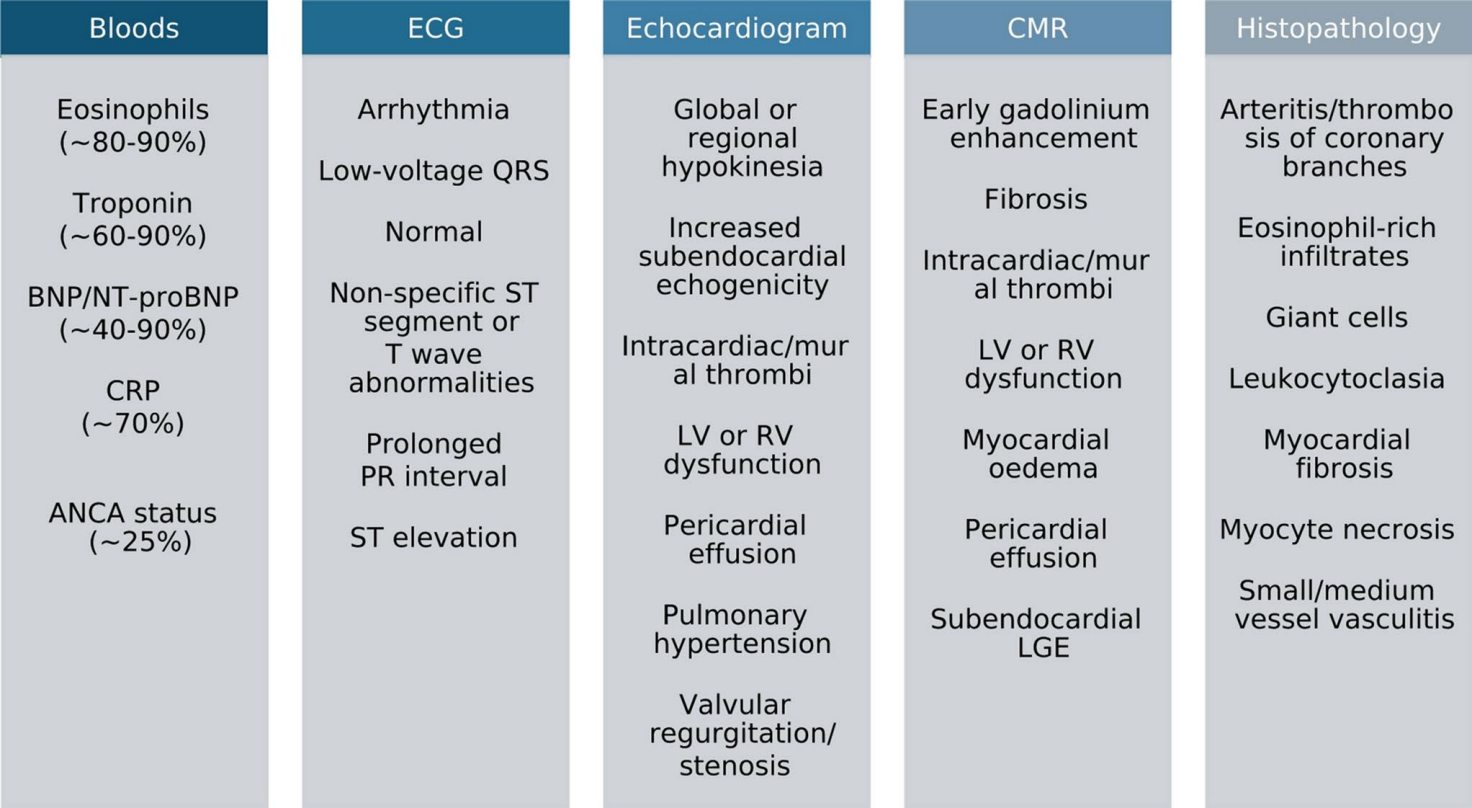
Cardiac investigations and potential findings in EGPA with heart involvement Diagnostic sensitivity of blood markers (%). Data derived from [8, 9, 22, 25, 30, 38]. Abbreviations: ANCA, Anti-neutrophil cytoplasmic antibodies; BNP, B-type natriuretic peptide; CMR, Cardiac magnetic resonance; CRP, C-reactive protein; ECG, Electrocardiogram; ESR, Erythrocyte sedimentation rate. LGE, Late gadolinium enhancement; LV, Left ventricle; NT-proBNP, N-terminal pro B-type natriuretic peptide; RV, Right ventricle

This section outlines key investigations (Fig. 3) to consider when screening for cardiac disease in patients with EGPA followed by a proposed diagnostic approach. It is important to note that the diagnostic criteria for cardiac disease in EGPA does vary across different studies, which may reflect differences in how diagnostic metrics are reported.

## Serum Biomarkers

### Eosinophil Count

Cardiac involvement in EGPA is strongly associated with eosinophilia. A meta-analysis by Brambatti et al. (2017), of 179 patients (of which 23 had EGPA) with histologically confirmed cardiac involvement, demonstrated that a raised peripheral blood eosinophil count was present in 90.9% of EGPA patients [22]. The study by Sartorelli et al. (2022) in 70 EGPA patients with cardiomyopathy, found a sensitivity of 78%, specificity of 35%, positive predictive value (PPV) of 46% and negative predicative value (NPV) of 69% for an eosinophil count of > 3000/mm^3^ [9]. However, during the acute stage of eosinophilic myocarditis, there are instances where peripheral eosinophilia is absent due to migration of eosinophils into the tissues and simultaneous eosinopoiesis in the bone marrow [13]. Furthermore, patients receiving treatments for eosinophilic asthma such as anti-IL-5/IL-5R biologics [mepolizumab, benralizumab) or high-dose glucocorticoids may not exhibit typical manifestations of peripheral eosinophilia. Measurement of eosinophilic specific mediators such as ECP and MBP is currently not validated [23].

### CRP

C-reactive protein (CRP) is a pentametric protein produced by the liver [24]. It is routinely performed in clinical practice and found to be elevated in response to inflammation. Elevated levels at presentation were found in 66.7% of EGPA patients with histologically proven myocarditis [22]. As a result, it may have limited effectiveness in screening for cardiac disease.

### ANCA

The archetypal association is the reporting of cardiac disease in ANCA-negative patients [5] and ANCA-positive patients tending to exhibit a vasculitis-like phenotype associated with glomerulonephritis and peripheral neuropathy, driven by ANCA and neutrophils [6]. However, there remains inconsistent reporting of this association [7–9]. The study by Sartorelli et al. [2022) of 70 EGPA patients with cardiomyopathy reported that 25% were ANCA-positive [9].

Although ANCA-positive patients are typically reported to have a lower prevalence of cardiac involvement [9], if patients test positive for ANCA, they should have periodic measurement of levels, as a rising tire may be indicative of a relapse. As ANCA-positive EGPA patients can still develop heart involvement, ANCA is an unreliable screening tool for detecting cardiac disease in EGPA.

### Cardiac Specific Troponin Assays

Raised serum concentrations of cardiac troponins may represent myocardial injury, inflammation, vascular damage or heart failure. Although non-specific, increased serum levels can support the diagnosis of cardiac involvement in EGPA patients. The meta-analysis from Brambatti et al. (2017) found troponin levels were elevated in 91.7% of patients [22]. The retrospective study by Sartorelli et al. (2022), found a sensitivity of 86%, specificity of 89%, PPV of 88% and NPV of 87% [9]. A recent study by Hua et al. (2024) used CMR to detect cardiac involvement in 84 EGPA patients. In this study, [26] patients were found to have myocardial EGPA, and the authors reported that troponin had an overall sensitivity of 56%, specificity of 66%, PPV of 41% and NPV of 78% [25]. Thus, CMR appears to be vastly superior to troponin for screening for cardiac disease in this cohort.

### Natriuretic Peptides

Natriuretic peptides such as BNP and NT-proBNP levels can be raised, reflecting left ventricular stretch, even in the context of mildly reduced or normal left ventricular (LV) systolic function on echocardiography. Sartorelli et al. (2022) report increased NT-pro-BNP measurements to have a sensitivity of 88%, specificity of 78**%**, PPV of 79% and NPV of 88% in diagnosing cardiomyopathy in EGPA [9]. Hua et al. (2024) report only a 38% sensitivity, 93% specificity, a PPV of 73% and NPV of 77% [25].

Raised natriuretic peptides can also be related to non-cardiac causes (such as sepsis, renal insufficiency, and cirrhosis) and thus a positive value should prompt further investigations to confirm the diagnosis of cardiac involvement in EGPA. Furthermore, a normal BNP can occur in instances of cardiac involvement. Despite their low sensitivity and specificity for diagnosis, serial measurements may be useful to assess treatment response alongside other first-line investigations when following up patients [5].

### Electrocardiography (ECG)

ECG abnormalities are reported in up to 66% of EGPA patients with cardiac involvement [5]. Findings may be non-specific, including indeterminate ST segment or T wave abnormalities, atrioventricular conduction disturbances, arrhythmias, pathological Q waves, complete and incomplete bundle branch blocks, and ST segment elevation in cases of coronary spasm or intracoronary thrombi [8, 26, 27]. However, given the high frequency (> 30%) of normal ECG findings in patients with cardiac involvement, additional investigations are recommended when screening for cardiac EGPA.

## Imaging

### Echocardiography

Current guidelines recommend obtaining an echocardiogram at the time of EGPA diagnosis, even in the absence of cardiac symptoms [28, 29]. Transthoracic echocardiography (TTE) may show LV dysfunction, intracardiac thrombi (typically found at the apex or subvalvular regions of atrioventricular valves with sparing of the outflow tracts), subendocardial echogenicity, increased LV wall thickness, pulmonary arterial hypertension and pericardial abnormalities. The right ventricle may also be involved, and abnormal diastolic function associated with high filling pressures may be evident [30]. Fibrotic valvular thickening and impaired subvalvular apparatus function may lead to valvular damage and atrioventricular valve regurgitation [30]. Stenotic lesions have also been described, including a case of severe mitral stenosis secondary to EGPA that resolved after systemic therapy [16]. TTE can also assist in assessing response to treatment and progression of disease and may detect features such as dilated or restrictive cardiomyopathy as a sequela of myocarditis [13].

A large series with 383 patients reported echocardiographic defects present in 27% of patients with EGPA [31]. Additionally, the meta-analysis by Brambatti et al. (2017), reported a median LV ejection fraction of 33% in 23 patients with EGPA myocarditis. Ventricular thrombus was reported in 19% of patients (typically LV), pericardial effusions were present in 27% and cardiac tamponade was present in one case [22]. A separate meta-analysis by Pakbaz et al. (2020) of 62 case reports of EGPA patients with cardiac involvement showed echocardiographic abnormalities in 96.8% of cases and evidence of pulmonary hypertension in 6.5% [8].

The diagnostic accuracy of conventional TTE may be improved with the use of contrast injection, helping delineate the shape of the left ventricle, hypertrophy and rule out apical thrombi [32]. Furthermore, transoesophageal echocardiography may allow better definition of valvular involvement and atrial thrombus detection [23].

### Cardiac Magnetic Resonance

With the advent of new multimodal diagnostic tools such as CMR, diagnosis and awareness of cardiac disease in EGPA has increased and this imaging modality now represents the non-invasive gold standard for diagnosis [13, 33].

CMR provides useful morphological and functional information in a variety of systemic and inflammatory diseases, and it is the most sensitive imaging technique for detection of inflammation, fibrosis and thrombosis. A meta-analysis assessing the Lake Louise Criteria (validated both clinically and using endomyocardial biopsy) to identify acute myocarditis (although not specific to EGPA) demonstrated a sensitivity of 80% and a specificity of 87% [34, 35].

In a meta-analysis of 62 EGPA patients with cardiac involvement, CMR was abnormal in all cases; all had elevated cardiac biomarkers, 33% had normal ECG findings, and two patients had normal echocardiography, highlighting that CMR may be necessary to define disease activity, extent, and severity in the heart [8]. Furthermore, in a recent retrospective analysis by Fijolek et al. (2023), 29% of patients (19 of 66 patients) had no clinical symptoms of heart disease, despite the presence of cardiac anomalies on CMR [36].

**Fig. 4:**
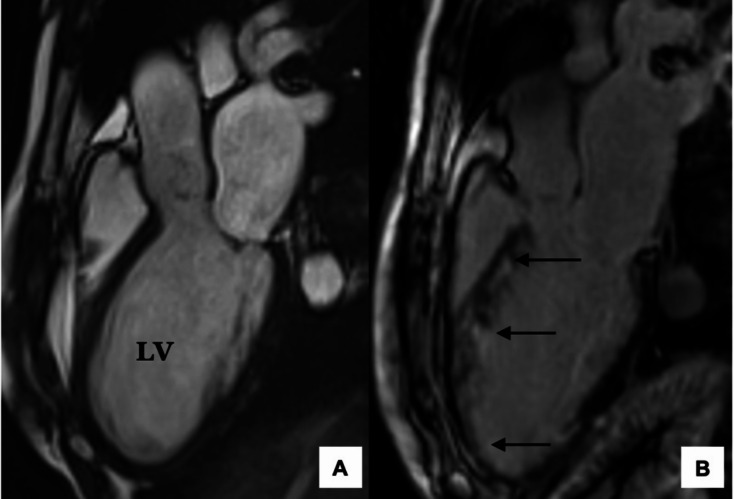
Subendocardial LGE distribution in EGPA. (**A**): 3 chamber view showing global LV dilatation. LV function was moderately impaired with LVEF of 35%. (**B**): There is subendocardial LGE along anterior wall, apex, and apical inferior wall (*arrows*). Abbreviations: LGE, Late gadolinium enhancement; LV, Left ventricular; LVEF, Left ventricular ejection fraction

Late gadolinium enhancement (LGE) has been established as the main sign of granulomatous inflammation on CMR in inflammatory disorders such as sarcoidosis [11] and has more recently been used for the identification of areas of active inflammation or fibrosis in patients with EGPA. CMR classically demonstrates a subendocardial LGE distribution (typically apical and mid-left ventricles) [37] (Fig. 4**)**. Myocardial LGE has been reported in 83–100% of patients in different studies in patients with symptomatic cardiomyopathy but also in up to a third of asymptomatic patients [11, 38]. This has raised questions about the diagnostic and prognostic significances of this feature in these patients [11]. A retrospective study of 42 patients, using LGE as the sole criterion for diagnosing cardiomyopathy, reported a sensitivity of 82% and a specificity of 56% [11]. Another study compared CMR results with endomyocardial biopsies and found that five out of six patients with LGE had endomyocardial fibrosis, thus demonstrating that LGE could reflect either acute inflammation and/or chronic fibrosis [38].

Differentiating active disease from fibrotic scars is pivotal in guiding treatment and there appears to be a limited role for immunosuppressive therapy in patients with asymptomatic LGE lesions without cardiomyopathy [11]. Therefore, while myocardial LGE is helpful in diagnosing cardiac involvement in EGPA, it should not be used as the sole diagnostic criterion.

CMR does provide other pertinent information on myocardial lesions such as T2-weighted signaling anomalies, extracellular volume quantification, specific topography, perfusion defects and segmental hypokinesia [11]. Multiparametric mapping, in addition to late (and early) gadolinium enhancement sequences can demonstrate specific markers of tissue damage such as capillary leakage, necrosis, fibrosis and intracellular/interstitial oedema [13]. In the acute phase, the presence of oedema appears hyperintense on T2-weighted sequences. Comparatively, in a chronic setting, fibrosis and necrosis (typically patchy and subendocardial in distribution), is detectable on LGE sequences. Thus T2-weighted myocardial signaling as a marker of oedema can assist in distinguishing acute from chronic myocardial damage [5].

**Fig. 5:**
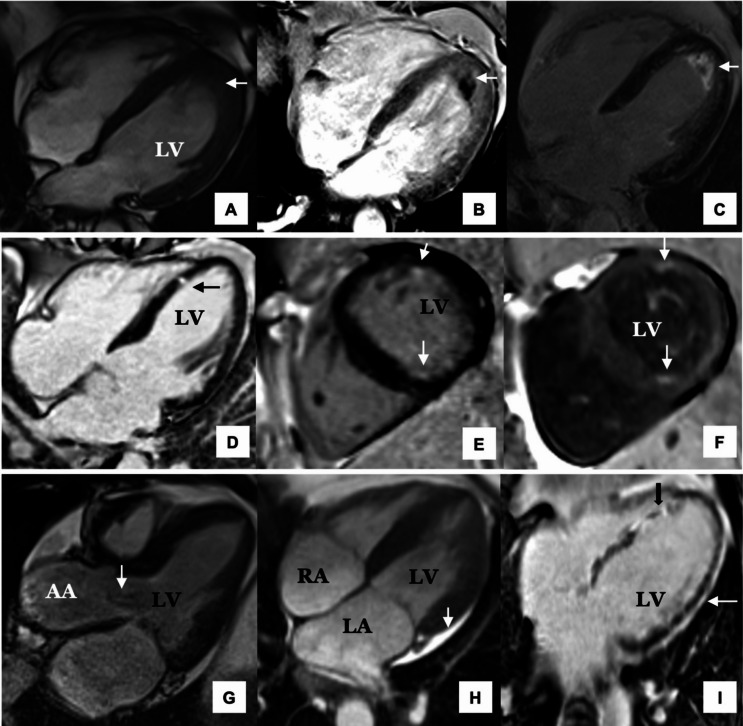
Cardiac pathology in EGPA. **A**-**C**: Thrombus– Cardiac MRI 4 chamber view showing **A**: thickening of the apex on the steady state free precision sequence (*arrow*). (**B**): In the same patient, the thrombus in the LV apex is better delineated on the early gadolinium sequence (*arrow*). (**C**): The same thrombus shows heterogenous LGE (*arrow*). (**D**-**F**): **A** series of images post gadolinium showing (**D**): multifocal tiny subendocardial infarcts along the septum (*arrow*). (**E**): Tiny subendocardial infarction inferior and anterolateral segments (*arrows*). (**F**): The infarcts (same patient from 3E) are better delineated on the black blood phase sensitive inversion recovery sequence (*arrows*). (**G**): Valvular disease– Steady state free precision sequence with the regurgitation jet across the aortic valve (*arrow*). (**H**): Chamber dilatation and Pericardial effusion– 4 chamber view steady state free precision sequence-left atrium (LA) is dilated. There is a small pericardial effusion (*arrow*). (**I**): Pericarditis– 4 chamber view shows intense subendocardial LGE along the interventricular septum (*black arrow*) and diffuse intense smooth pericardial enhancement (*white arrow*) in keeping with pericarditis. Abbreviations: LA, Left Atrium; LGE, Late gadolinium enhancement; LV, Left ventricular; MRI, magnetic resonance imaging

In addition to echocardiography, CMR can effectively detect alternative pathology found in cardiac EGPA such as pericarditis, pericardial effusion, thrombus and valvular disease (Fig. 5).

**Fig. 6:**
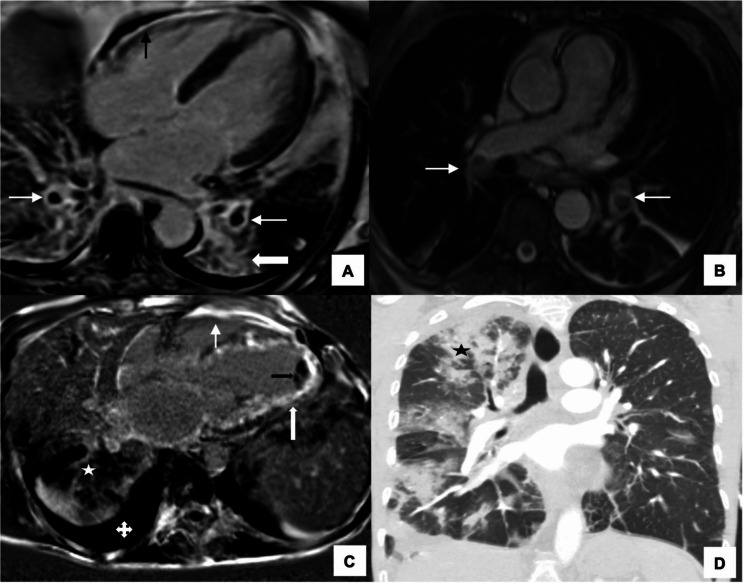
Lung disease associated with cardiac EGPA. **A**-**B**: Pulmonary embolism and pericarditis. **A**: CMR 4 chamber view shows bilateral segmental PE in both lower lobes (*thin white arrows*) with basal pulmonary infarction (*block arrow*). There is also intense smooth pericardial enhancement in keeping with pericarditis (*black arrow*). **B**: Axial images show bilateral pulmonary emboli within the lobar right and left lower lobe pulmonary arteries (*arrows*). **C**-**D**: Biventricular involvement with pulmonary infiltrates. **C**: 4 chamber CMR imaging in a 40-year-old with EGPA shows diffuse biventricular LGE (right ventricle, *thin arrow*; left ventricle, *block arrow*) with a LV apical thrombus (*black arrow*). There is ill-defined right lung pulmonary consolidation (*star*) and pleural effusion (*quad arrow*). **D**: The corresponding CT thorax better delineates the pulmonary parenchymal abnormality (*star*). Abbreviations: CMR, Cardiac magnetic resonance; CT, Computed tomography; EGPA, eosinophilic granulomatosis with polyangiitis; LGE, Late gadolinium enhancement; LV, left ventricle; PE, Pulmonary embolism

Finally, it is not uncommon to detect non-cardiac pathology on CMR such as pulmonary infiltrates secondary to EGPA lung disease and pulmonary emboli (Fig. 6).

## Nuclear Medicine Techniques

Nuclear imaging techniques such as Fluoro-2-deoxyglucose positron emission tomography (FDG-PET) and Gallium-67 (^67^Ga) scintigraphy have been used in the diagnosis and follow-up of cardiac involvement in other autoimmune disorders such as cardiac sarcoidosis, systemic lupus erythematosus and Kawasaki disease [30, 39, 40].

### Fluoro-2-Deoxyglucose PET

**Fig. 7:**
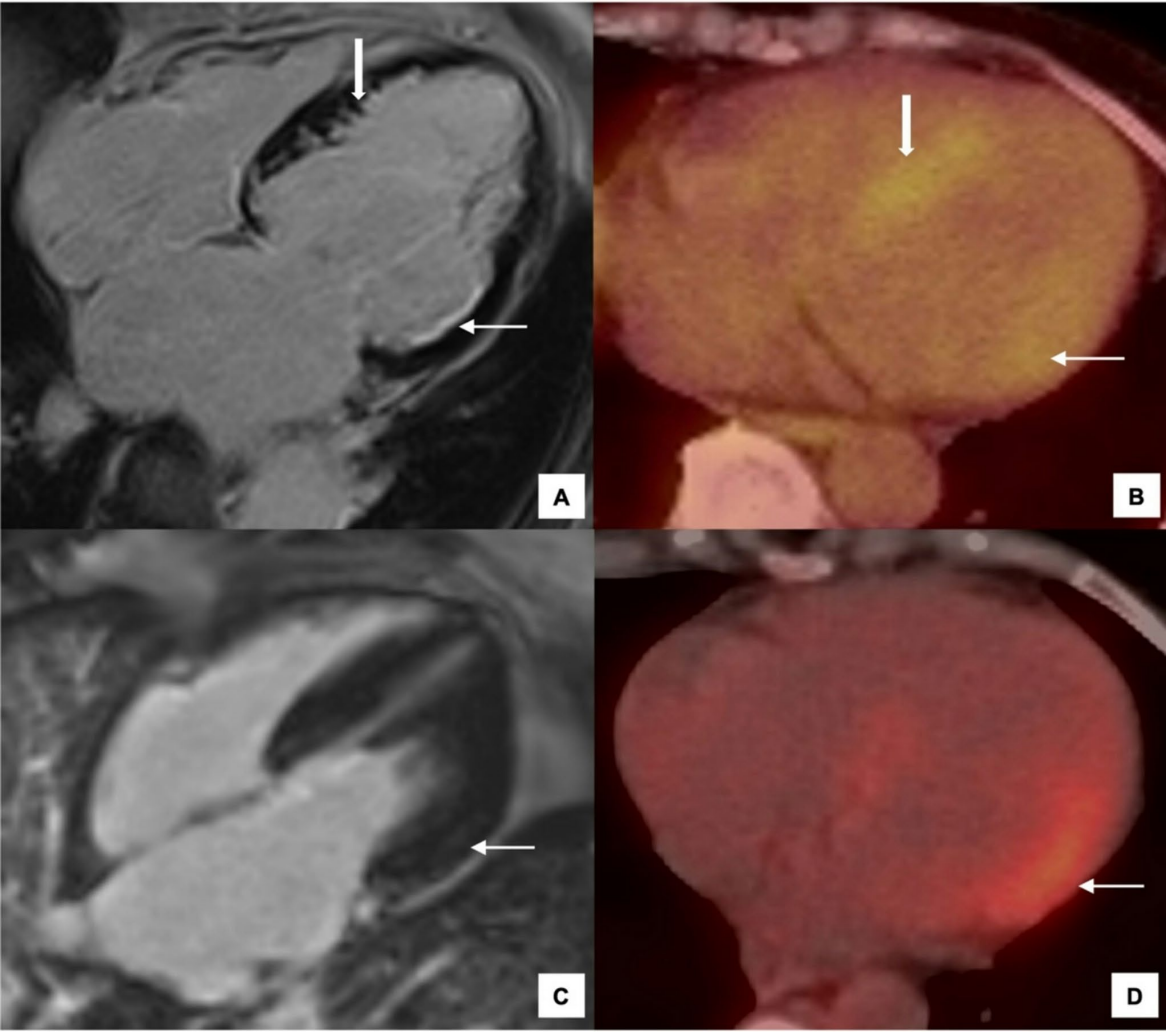
Use of FDG-PET imaging in EGPA. **A**-**B**: Example of active inflammation & scarring on PET. **A**: 4 chamber cardiac MRI shows subendocardial LGE in the basal lateral wall (*thin arrow*) and along the septum (*block arrow*). **B**: The corresponding FDG PET-CT demonstrates increased metabolic activity in the septum (*block arrow*) in keeping with active inflammation while there is only subtle increase in FDG uptake along the basal lateral wall (*thin arrow*) suggestive of established myocardial scarring. **C**-**D**: Increased sensitivity of PET to pick up active inflammation. **C**: 4 chamber cardiac MRI shows subtle LGE (*arrow*) in the basal lateral wall. **D**: FDG-PET-CT shows intense increased metabolic activity (*arrow*) in the basal lateral wall. Abbreviations: CT, Computed tomography; FDG, Fluoro-2-deoxyglucose; LGE, Late gadolinium enhancement; PET, positron emission tomography

FDG-PET with cardiac gating has been used as a non-invasive imaging modality for the detection of cardiac involvement in EGPA (Fig. 7) and can provide complimentary information to CMR. In fact, active inflammation can occasionally be detected by FDG-PET in instances where CMR shows only subtle LGE (Fig. 7). A study by Marmursztejn et al. (2013) demonstrated that in patients who were in clinical remission from EGPA, 14 of 20 (70%) showed signs of myocardial involvement when evaluated by CMR [41]. This was hypothesised to be related to myocardial fibrosis rather than ongoing active inflammation. On FDG-PET, 10 of 14 (71%) had low FDG uptake, suggesting myocardial fibrosis accounted for CMR findings, two patients had high uptake, reflecting active inflammation, and two patients had normal FDG uptake. Thus, of the 14 patients with abnormal CMR imaging, two patients showed signs of acute inflammation on FDG-PET, indicating that active myocardial inflammation may persist in patients who are otherwise considered to be in disease remission [41]. However, this should be contextualised with the important caveat that increased FDG uptake is not necessarily specific to inflammation. There are also technical limitations of FDG and possible artifacts related to an improper diet preparation. Furthermore, since the inception of the study, there have been advances in CMR techniques.

Distinguishing between acute and chronic lesions is important to prevent unnecessary administration of systemic therapy and nuclear medicine techniques may assist in this distinction, however there are currently limited data specific to patients with EGPA to make definitive recommendations. PET scan may also detect improvements prior to echocardiographic findings when following up patients [42]. Therefore, our view is that cardiac-gated FDG-PET may be considered as an alternative diagnostic imaging technique, for example in those who have a contraindication/intolerance to CMR or if CMR findings are equivocal.

### ^67^Ga scintigraphy

^**67**^Ga Scintigraphy has been used in some cases of inflammatory cardiomyopathies [43]. However, it is met with varying results in terms of diagnosing and monitoring cardiac involvement in these patients [40, 43]. There has been limited use of ^**67**^Ga Scintigraphy in patients with EGPA with cardiac involvement and it is not routinely performed.

### Coronary Angiography

Coronary artery involvement in patients with EGPA is rare (estimated < 7%) [22, 44]. Coronary angiography may show stenotic lesions, coronary ectasia, or vasospasm with the latter diagnosis being the main cause of chest pain in EGPA patients without significant atherosclerotic coronary disease. Coronary angiography (or in some instances, non-invasive coronary CT) is recommended in acute coronary syndrome-like presentations, including ST segment elevation on ECG, raised cardiac troponin or wall motion abnormalities on imaging [13].

### Endomyocardial Biopsy

The invasive diagnostic gold standard for suspected inflammatory cardiac diseases is endomyocardial biopsy (EMB) [13, 30] as it provides conclusive histopathological proof of eosinophilic myocarditis. However, its use in clinical practice has now diminished given risks of the procedure and improved access to non-invasive imaging such as CMR. In addition, unlike CMR, EMB has limited sensitivity (around 50%), owing to the focal and patchy nature of inflammatory damage [45]. However, EMB may be useful to confirming EGPA diagnosis and rule out other causes of inflammatory cardiac diseases when there is diagnostic uncertainty. EMB may detect inflammation when imaging has proven inconclusive, and it is the only method able to define the histological subtypes of cardiac inflammation [39].

The main histological pattern is an eosinophil-rich infiltrate of the myocardium. EMB can also show more specific findings such as eosinophilic granulomata, fibrinoid necrosis and small and medium-sized vessel vasculitis [30]. Immunohistochemistry and immunofluorescence for eosinophil-specific proteins may also allow better characterization of cell infiltrates [23]. EMB, however, is not without risk and absolute contraindications include acute myocardial infarction, left ventricular thrombosis and ventricular aneurysm [13]. The decision to perform EMB should be patient-centered after careful risk-benefit analysis [23].

### Diagnostic Approach

**Fig. 8:**
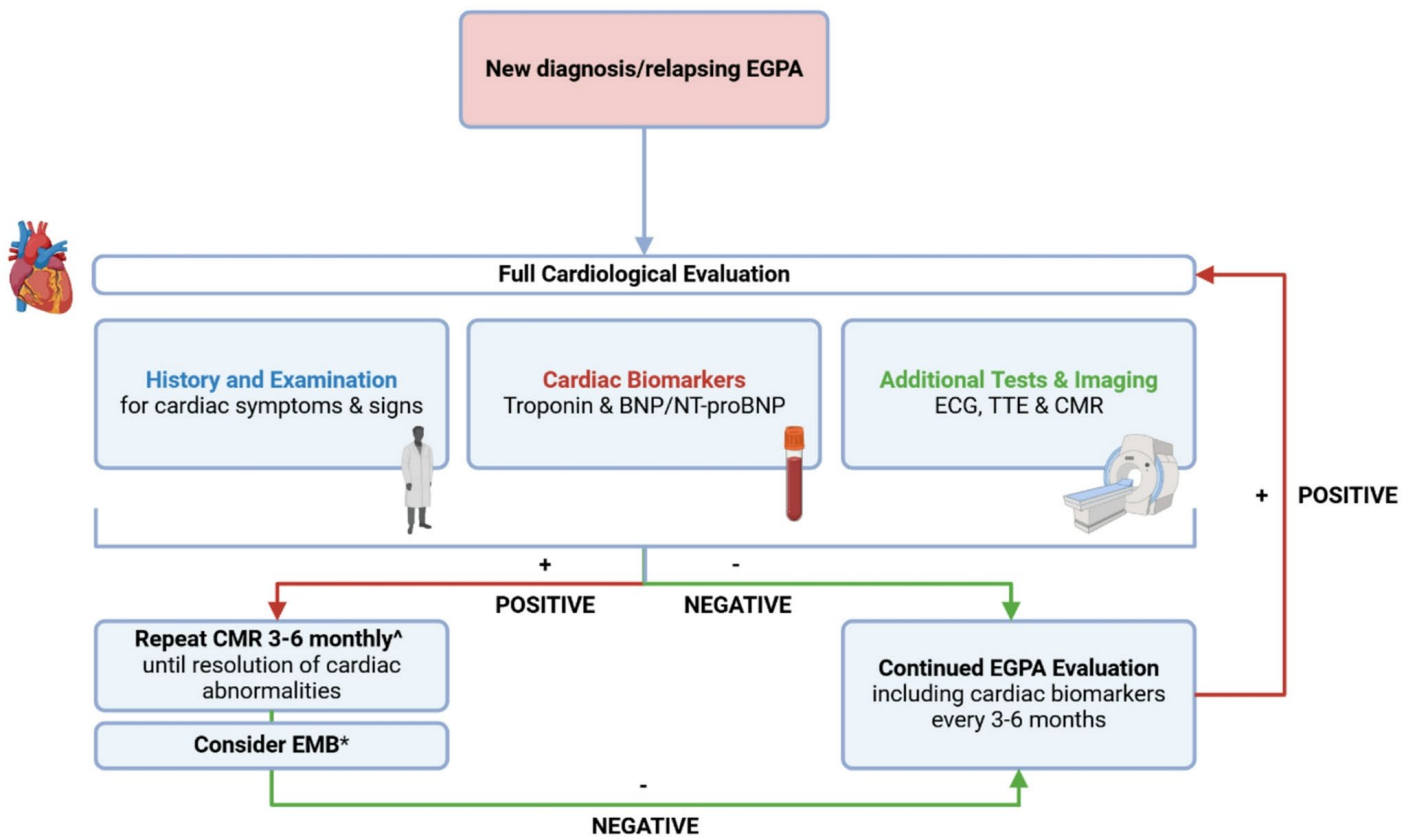
Proposed algorithm for the evaluation of cardiac involvement in EGPA. We recommend all newly diagnosed/relapsing EGPA patients to have first-line cardiological screening assessments: BNP/NT-proBNP, troponin, ECG, TTE and CMR. ^If positive findings are found on initial CMR, repeat CMR is useful at 3–6 months 48. Frequency of further surveillance imaging should be tailored to the individual and requires the input of the multidisciplinary team. Cardiac-gated FDG-PET may be considered as an alternative/adjunctive diagnostic imaging technique in those who have contraindication/intolerance to CMR, or in those in whom uncertainty remains. *Consider EMB for histopathological diagnosis dependent on patient preference and local expertise. All EGPA patients should have periodic assessment of cardiac biomarkers. Abbreviations: BNP, B-type natriuretic peptide; CMR, cardiac magnetic resonance; ECG, electrocardiogram; EGPA, eosinophilic granulomatosis with polyangiitis; EMB, endomyocardial biopsy; NT-proBNP, N-terminal B-type natriuretic peptide; TTE, transthoracic echocardiography. Figure created in BioRender. Wilson-Morkeh, H. (2025) https://BioRender.com/4qjjt51

Given the high incidence and poor prognosis of EGPA-related cardiac disease, careful cardiological work-up (Fig. 8) is warranted for all patients at diagnosis of EGPA and a multi-disciplinary team approach is required [11]. When investigating for cardiac disease, distinguishing active inflammation from chronic scar is essential as this has important treatment implications for the patient.

Baseline cardiac history, examination and investigations should be performed in all patients with EGPA [7]. Those with cardiac symptoms at presentation will usually have these investigations per indication; however, since a significant proportion (typically 30–45%) of patients do not display cardiac symptoms [5], we recommend these first-line screening assessments in all patients regardless of symptoms: BNP/NT-proBNP, troponin, ECG, TTE and CMR (Fig. 8**)**. If CMR is not possible (or is equivocal), FDG-PET can be considered and potentially downstream tests such as coronary angiography depending on the clinical context [30].

### Should all Patients Undergo CMR Imaging?

Current guidelines recommend periodic echocardiography and ECG for all EGPA patients to facilitate early detection of asymptomatic cardiac involvement, while CMR is advised only for patients with overt cardiomyopathy [28, 46].

However, there may be situations where patients are asymptomatic with negative findings on first-line investigations. CMR for example, has demonstrated a high prevalence of cardiac abnormalities in the absence of clinical symptoms, ECG or echocardiographic findings [47]. The recent study by Hua et al. (2024) diagnosed cardiac disease using CMR (as part of baseline clinical evaluation) in almost one-third of their EGPA patients. The authors suggest that CMR should be included in the baseline evaluation of all EGPA patients, due to the high prevalence of cardiac involvement in asymptomatic patients [25].

Conversely, another study by Fijolek et al. (2023) found that CMR-detected anomalies did not necessarily adversely affect the prognosis and outcomes for asymptomatic patients with no other signs of cardiomyopathy [36]. As imaging techniques become more sensitive, there is a concern that we may end up detecting non-significant pathology, potentially leading to overtreatment.

This remains a contentious area but nevertheless important as there is a risk of overlooking asymptomatic patients who may be at risk of fatal cardiac events or could develop irreversible cardiac damage due to fibrosis. In a large retrospective review by Comarmond et al. (2013), out of 383 patients, they reported 14 fatal cardiac events with only eight reported in patients with known EGPA-related cardiomyopathy. Our view is that given the non-invasive nature and lack of ionising radiation with magnetic resonance imaging, all patients should undergo CMR at EGPA diagnosis (in centres that have access to this).

## Follow-up

### Cardiac Biomarkers

We recommend repeating cardiac biomarkers (troponin and BNP/NT-proBNP) every 3–6 months in all patients with EGPA (Fig. 8) and this may be more frequent in those with cardiac disease depending on the clinical context.

### Imaging

Following initiation of treatment for cardiac disease, repeat CMR is also advised to assess for any ongoing cardiac inflammation.

Follow up CMR, while not always able to precisely identify the nature of remaining lesions, offers valuable insight into which lesions may lead to future cardiac complications. For example, improvement of myocardial LGE on follow-up CMR is significantly associated with the absence of new cardiac complications. Alternatively, the worsening or long-term stabilization of LGE was significantly associated with the advent of major cardiac complications [9, 11].

In our experience [48], repeat CMR at 3–6 months (depending on the duration of induction treatment) is an appropriate time interval to detect improvements in inflammation (Fig. 8). More frequent and further follow up imaging should be tailored to the individual patient and will require a multi-disciplinary team approach.

## Treatment

Active myocarditis in patients with EGPA warrants urgent systemic immunosuppressive therapy to prevent acute disease complications (arrhythmia, heart failure) and to mitigate progression to myocardial fibrosis, LV systolic dysfunction and irreversible myocardial damage [39]. To date, there is no specific regimen that has been tested for treatment of cardiac disease in EGPA in clinical trials.

Recently published evidence-based guidelines [46], British Society for Rheumatology (BSR) [49], European Alliance of Associations for Rheumatology (EULAR) [50], American College of Rheumatology (ACR) [28] and the European EGPA study group [51] state that patients with organ-threatening manifestations included in the Five-Factor score should be treated with glucocorticoids (GC) as initial therapy followed by cyclophosphamide (CYC) or rituximab (RTX).

### Glucocorticoids

Although with limited evidence base, guidelines advise the use of pulsed intravenous GC (typically daily methylprednisolone pulses of 500–1000 mg daily over three days, maximum total dose of 3 g) in the case of life-threatening cardiac manifestations [46]. Following this, high dose oral glucocorticoids (e.g. 0.75-1 mg/kg per day) are recommended, followed by a slow tapering regimen.

Following initial GC therapy, we typically observe a rapid reduction in blood eosinophils and troponin levels, with reduction in myocardial inflammation and symptoms [52, 53]. However, a prompt response in these markers should not preclude proceeding with definitive immunosuppression.

### Cyclophosphamide

The use of CYC in other forms of vasculitis (such as granulomatosis with polyangiitis (GPA) and microscopic polyangiitis (MPA) [54], has made it a popular choice for remission induction in patients with active, severe EGPA. Its use has been particularly favoured for those who are ANCA-negative, or with cardiac, severe gastrointestinal or neurological involvement [28]. The difference between intravenous over oral use is unknown in EGPA but a randomized controlled trial (RCT) of 149 GPA and MPA patients showed that there was no clear difference in remission outcomes. However, oral administration was associated with a greater cumulative dose and increased risk of leukopaenia, infection and malignancy [55]. We therefore recommend intravenous CYC (according to local dosing protocols for severe EGPA), over oral administration in patients with cardiac involvement.

The optimum duration of CYC induction therapy in these patients remains to be established. In an RCT of patients with EGPA and FFS≥1 (27 patients in total with cardiac involvement), twelve pulses of CYC had a slightly longer relapse-free survival than six pulses but did not reduce severe relapses in all patients [56]. We recommend that CYC induction should be continued until remission is achieved, typically within 6 months. For those who are slower to improve, and do not reach complete remission by 6 months, longer induction periods may be needed (in the case of cardiac EGPA, this may be guided by repeat CMR).

### Rituximab

Increasing use of RTX for GPA/MPA has led to more patients with severe EGPA receiving RTX (particularly those who are ANCA-positive, with active glomerulonephritis or prior CYC exposure). In 2010, two landmark randomized controlled trials, Rituximab for ANCA-associated Vasculitis (RAVE) and Randomized Trial of Rituximab versus Cyclophosphamide for ANCA-associated Renal Vasculitis (RITUXVAS) studies, demonstrated that RTX was non-inferior to treatment with CYC for induction treatment with AAV, however these studies did not include patients with EGPA. The data on RTX use in EGPA is limited and mainly confined to those who have relapsing disease [58].

Recent studies have demonstrated efficacy of RTX in EGPA patients with cardiac disease [59–61]. For example, Teixeira et al. (2019] reported some efficacy of use of RTX in an EGPA cohort of 69 patients, 21% of whom had cardiac disease [60]. Patients received RTX either as 1 g doses administered two weeks apart or as 375 mg/m^2^ weekly for four weeks. They reported improvements in the Birmingham Vasculitis Activity Score (BVAS) and reductions in prednisolone dose to a median of 5 mg at 24 months in all patients. Mohammad et al. (2014) reported efficacy with RTX with reduction in requirement of steroids in 41 patients with EGPA, 22% of which had cardiac involvement [61]. The Rituximab in Eosinophilic Granulomatosis with Polyangiitis (REOVAS) RCT (published in abstract form in 2021), also showed that RTX was comparable with CYC for induction remission in patients with an FFS ≥ 1, however no strong conclusions have been made regarding non-inferiority [62].

### Combination Therapy

The combination of RTX and low-dose CYC has emerged as a potential remission induction strategy for patients with severe GPA/MPA, that may provide rapid disease control and enable GC avoidance [57, 63–65]. At our centre, we typically administer two doses of RTX 1 g (day 0 and week 2) and six intravenous doses of CYC 500-750 mg (day 0 and weeks 2, 4, 6, 8 and 10) in these patients. Although controlled evidence of this regimen in patients with EGPA is lacking, we have locally translated this approach to patients with severe EGPA, with the aim of reducing cumulative exposure to CYC and GC through the addition of RTX. This is particularly pertinent in EGPA as long-term GC dependence is common, largely driven by refractory asthma and allergic disease [66]. Initial findings suggest that it may be effective in patients with EGPA and cardiac involvement with a favourable safety profile [67].

### Anti-IL-5 Therapies

The drivers of cardiac disease include infiltrating eosinophils, and an ideal therapy would be targeted with limited off-target side effects. IL-5 is a key cytokine produced by eosinophils, playing an important role in the proliferation, maturation, activation, recruitment and survival of these cells [68]. Thus, blockade of IL-5 or the IL-5 receptor α (IL-5Rα) are therapies which have garnered recent interest [69].

Mepolizumab is a humanised monoclonal antibody that binds to and neutralises IL-5 [3). It has been approved for severe asthma and EGPA based on the Mepolizumab In Relapsing or Refractory EGPA (MIRRA) study, the first phase III RCT for EGPA, however patients with organ or life-threatening manifestations at time of study entry were excluded [70]. Additionally, a multicentre retrospective study reported good efficacy with mepolizumab in EGPA patients with cardiac involvement [71]. At 24 months, nine out of ten patients who had cardiac disease achieved full remission, whilst just one patient had persisting cardiac disease. However, EGPA-related myocarditis has been reported whilst on mepolizumab treatment, which may suggest some involvement of vasculitis activity, in addition to IL-5 driven eosinophilic injury in these patients [72].

Benralizumab is an anti-IL-5Rα monoclonal antibody which prevents IL-5 from binding its receptor, inhibiting the maturation and survival of eosinophils, in addition to inducing apoptosis of eosinophils via antibody-dependent cellular cytotoxicity [73]. It was found to be non-inferior to mepolizumab in the MANDARA study [74]; however, this study also excluded patients with organ or life-threatening EGPA within 3 months of the first visit. Nevertheless, it has been shown to be effective in an EGPA patient diagnosed with Staphylococcus aureus sepsis with cardiac involvement [75], a patient with recurrent cardiac arrest due to eosinophilia-related coronary vasospasm [76] and a patient presenting with a cardiac relapse whilst on RTX maintenance treatment [77].

In summary, there is currently insufficient evidence to suggest use of anti-IL-5 therapies in EGPA patients with cardiac disease [78, 79]. However, in certain situations, for example where standard of care is contraindicated or following relapse, they may play a role. These treatments may assist in gaining rapid control of eosinophilia and can also be used as a steroid-sparing strategy during maintenance therapy. Furthermore, targeting both vasculitic and eosinophilic inflammation as a combination approach (i.e. with CYC or RTX) could be effective. There are case reports of the combination of RTX and mepolizumab being effective in cardiac disease in EGPA [80, 81], although clinical trials are required to address an optimal treatment regimen.

### Intravenous Immunoglobulin

There are limited data on EGPA patients with cardiac involvement receiving treatment with intravenous immunoglobulin (IVIG) [3]. Case reports describe an increase in left ventricular ejection fraction and recovery of myocardial function in a small number of patients with cardiac involvement [82–84], however further studies are required to confirm the effectiveness of this therapy.

### Antithrombotic Therapy

Patients with EGPA and cardiac disease often suffer with acute thrombosis, which may be arterial or venous (typical association is an LV thrombus). These should be treated with anticoagulation, anti-platelet or thrombolytic agents even in the context of active vasculitis being the driver of pathology. It is generally accepted that the duration of anticoagulation should be tailored to disease activity, although data supporting this is lacking [23]. Thrombosis prophylaxis in patients with cardiac involvement should be guided by individual risk assessment, with a low threshold for initiation in hospitalised patients.

### General Cardiovascular Health Management

An important cause of cardiovascular disease in EGPA patients is accelerated atherosclerosis resulting from systemic inflammation [85]. Patients should have cardiovascular health optimised including management of cardiovascular risk factors. Those with heart failure should be managed according to general recommendations on heart failure [86].

We recommend periodic screening of classical cardiovascular disease (CVD) risk factors including hypertension, dyslipidaemia and glucose intolerance, factors which can result from the disease itself but also due to off-target treatment side effects. Lifestyle modification should include smoking cessation, weight control, healthy eating habits and physical activity, the level of which should be individually tailored depending on baseline level of function [87]. However, physical activity has been advised to be restricted during the acute phase of myocardial inflammation, and this may include the first 6 months of treatment [88].

### Limitations and Future Strategies

Current immunosuppressive therapies are effective in combatting inflammation; however long-term GC use is linked to cardiovascular disease and CYC itself can be complicated by cardiotoxicity [89]. Although rare, RTX can also be associated with adverse cardiac side effects such as arrhythmia and non-ischaemic cardiomyopathy, particularly in patients with a prior cardiovascular history [90, 91].

No treatments have been studied specifically for EGPA and heart disease in humans. However, a study conducted in mice has revealed the importance of eotaxins and chemokine receptor CCR3 for eosinophil migration and localization to the heart [92]. Furthermore, another study showed mice deficient in interferon-γ and IL-17 A developed fatal eosinophilic myocarditis, suggesting protective effects of these cytokines in eosinophilic heart disease [93].

The genome-wide association study (GWAS) of EGPA identified variants in the gene encoding thymic stromal lymphopoietin (TSLP) [94]. This alarmin, an endogenous constitutively expressed protein released in response to stimuli, is reported to be elevated in the serum of individuals with active EGPA, in addition to IL-25 and soluble ST2 (sST2), the decoy receptor of IL-33 [95]. These alarmins potentiate IL-5 production upstream via activation of innate lymphoid type-2 cells (ILC2s), thus offering an alternative potential treatment pathway.

Avacopan is a small-molecule C5a receptor antagonist approved for use in severe GPA and MPA following positive results in the Avacopan for the Treatment of ANCA-associated vasculitis (ADVOCATE) trial [96]. There is no data for the efficacy of avacopan in EGPA as the trial excluded such patients; however, its potential role in severe EGPA disease is attractive given the presence of the C5a receptor on eosinophils and its role in eosinophil effector functions [97, 98]. Clinical trials are warranted to explore this potential future therapeutic avenue.

## Conclusions

Cardiac disease in EGPA can present with a wide spectrum of symptoms and pathologies. Given its serious prognostic implications, careful cardiological work up is required. Recent consensus guidelines have appreciated the importance of electrocardiography and echocardiography; however, CMR is the only non-invasive diagnostic tool that can reliably produce information on early cardiovascular involvement in patients with EGPA. We propose baseline CMR to be performed in all patients at EGPA diagnosis, but long-term studies are required to understand if periodic CMR is of any benefit. Timely administration of appropriate systemic immunosuppressive therapy with glucocorticoids and cyclophosphamide is crucial to prevent both acute and long-term cardiac complications, and generally, good rates of cardiac remission have been observed [8, 48]. The biologics era has led to an increase in the use of anti-IL-5 therapies and RTX, which have both demonstrated promising cardiac outcomes in EGPA whilst exhibiting potential to reduce the significant long-term glucocorticoid dependence in these patients. Larger prospective studies and RCTs are required to further assess their efficacy, identify more reliable diagnostic biomarkers and test novel treatment targets for improved clinical outcomes.

## Key References

- Hua A, Sularz A, Wheen P, Alam V, Rajani R, Chiribiri A, et al. Detection of Cardiac Involvement in Eosinophilic Granulomatosis With Polyangiitis (EGPA) With Multiparametric Cardiovascular Magnetic Resonance (CMR). JACC Cardiovasc Imaging. 2024 Jun 24.
  - Findings from this study suggests that CMR should be considered as part of the baseline evaluation for all patients with EGPA.
- Sartorelli S, Chassagnon G, Cohen P, Dunogué B, Puéchal X, Régent A, et al. Revisiting characteristics, treatment and outcome of cardiomyopathy in eosinophilic granulomatosis with polyangiitis (formerly Churg–Strauss). Rheumatology. 2022 Mar 2;61(3):1175–84.
  - Findings from this study suggests that CMR should be considered as part of the baseline evaluation for all patients with EGPA.

## Data Availability

No datasets were generated or analysed during the current study.
